# Supplementary material for: Characterization of VvmiR166s-Target Modules and Their Interaction Pathways in Modulation of Gibberellic-Acid-Induced Grape Seedless Berries
Source: Int J Mol Sci. 2023 Nov 14;24(22):16279. doi: 10.3390/ijms242216279 (PMC10670991; doi:10.3390/ijms242216279)
Supplement: Supplementary file 1 [file ijms-24-16279-s001.zip › Table S1.pdf]

Table S1 Target gene prediction of VvmiR166s

miRNA_Acc	miRNA_Length	Expectation	UPE	Target_Acc.	Target_Regions	Inhibition
TCGGACCAGGCGTTCATTCC	20	3	22.742	*VIT_204s0008g03250.1*（*HB-15*）	CDS:1205-1223	Cleavage
	20	3	22.742	*VIT_204s0008g03250.2*（*HB-15*）	CDS:1205-1223	Cleavage
	20	3	21.088	*VIT_210s0003g04670.1*（*HOX32*）	CDS:1065-1083	Cleavage
	20	3	21.088	*VIT_210s0003g04670.2*（*HOX32*）	CDS:431-449	Cleavage
	20	3	23.076	*VIT_210s0003g04670.3*（*HOX32*）	CDS:1159-1177	Cleavage
	20	3	23.995	*VIT_213s0019g04320.1*（*REVOLUTA-like*）	CDS:1114-1132	Cleavage
	20	3	23.995	*VIT_213s0019g04320.2*（*REVOLUTA-like*）	CDS:1114-1132	Cleavage
	20	3	23.995	*VIT_213s0019g04320.3*（*REVOLUTA-like*）	CDS:1114-1132	Cleavage
	20	3	23.995	*VIT_213s0019g04320.4*（*REVOLUTA-like*）	CDS:444-462	Cleavage
	20	3	23.995	*VIT_213s0019g04320.5*（*REVOLUTA-like*）	CDS:387-405	Cleavage
	20	3	23.995	*VIT_213s0019g04320.6*（*REVOLUTA-like*）	CDS:1114-1132	Cleavage
	20	3	23.995	*VIT_213s0019g04320.7*（*REVOLUTA-like*）	CDS:1114-1132	Cleavage
	20	3	22.12	*VIT_206s0004g02800.1*（*REVOLUTA*）	CDS:1322-1340	Cleavage

	20	3	22.12	*VIT_206s0004g02800.2（REVOLUTA）*	CDS:1730-1748	Cleavage
	20	3	22.12	*VIT_206s0004g02800.3（REVOLUTA）*	CDS:1746-1764	Cleavage
	20	3	18.711	*VIT_203s0097g00550.1*	CDS:919-938	Translation
TCGGACCAGGCTTCATTCCCC	21	2	22.742	*VIT_204s0008g03250.1（HB-15）*	CDS:1204-1223	Cleavage
	21	2	22.742	*VIT_204s0008g03250.2（HB-15）*	CDS:1204-1223	Cleavage
	21	2	21.088	*VIT_210s0003g04670.1（HOX32）*	CDS:1064-1083	Cleavage
	21	2	21.088	*VIT_210s0003g04670.2（HOX32）*	CDS:430-449	Cleavage
	21	2	23.076	*VIT_210s0003g04670.3（HOX32）*	CDS:1158-1177	Cleavage
	21	2	23.995	*VIT_213s0019g04320.1（REVOLUTA-like）*	CDS:1113-1132	Cleavage
	21	2	23.995	*VIT_213s0019g04320.2（REVOLUTA-like）*	CDS:1113-1132	Cleavage
	21	2	23.995	*VIT_213s0019g04320.3（REVOLUTA-like）*	CDS:1113-1132	Cleavage
	21	2	23.995	*VIT_213s0019g04320.4（REVOLUTA-like）*	CDS:443-462	Cleavage
	21	2	23.995	*VIT_213s0019g04320.5（REVOLUTA-like）*	CDS:386-405	Cleavage
	21	2	23.995	*VIT_213s0019g04320.6（REVOLUTA-like）*	CDS:1113-1132	Cleavage
	21	2	23.995	*VIT_213s0019g04320.7（REVOLUTA-like）*	CDS:1113-1132	Cleavage

21	2	22.12	*VIT_206s0004g02800.1*（*REVOLUTA*）	CDS:1321-1340	Cleavage
21	2	22.12	*VIT_206s0004g02800.2*（*REVOLUTA*）	CDS:1729-1748	Cleavage
21	2	22.12	*VIT_206s0004g02800.3*（*REVOLUTA*）	CDS:1745-1764	Cleavage
21	2.5	20.995	*VIT_218s0075g00480.1*	CDS:3094-3113	Translation
21	2.5	20.995	*VIT_218s0075g00480.2*	CDS:3094-3113	Translation
21	2.5	20.995	*VIT_218s0075g00480.3*	CDS:2850-2869	Translation
21	2.5	20.995	*VIT_218s0075g00480.4*	CDS:2830-2849	Translation
21	2.5	20.995	*VIT_218s0075g00480.5*	CDS:3094-3113	Translation
21	2.5	20.995	*VIT_218s0075g00480.6*	CDS:3327-3346	Translation
21	2.5	20.995	*VIT_218s0075g00480.7*	CDS:2066-2085	Translation
21	3	18.46	*VIT_216s0050g01140.1*	CDS:357-376	Translation
21	3	17.375	*VIT_208s0007g03420.1*	CDS:364-383	Cleavage

The sequences of miRNAs and their target genes were compared by BioXM software to predict the mismatch numbers and the target regions.
