# Supplementary material for: Characterization of VvmiR166s-Target Modules and Their Interaction Pathways in Modulation of Gibberellic-Acid-Induced Grape Seedless Berries
Source: Int J Mol Sci. 2023 Nov 14;24(22):16279. doi: 10.3390/ijms242216279 (PMC10670991; doi:10.3390/ijms242216279)
Supplement: Supplementary file 1 [file ijms-24-16279-s001.zip › Table S2.pdf]

Table S2 Functional annotation of predicted target genes

Target gene		Interval	length	E-value	Target gene annotation
VIT_206s0004g02800	*REVOLUTA*	8460-8621	161	6.51E-13	homeobox leucine-zipper protein
VIT_213s0019g04320	*REVOLUTA-like*	2456-2617	161	6.04E-13	homeobox-leucine zipper protein revoluta
VIT_204s0008g03250	*HB-15*	8733-8897	164	1.61E-12	class iii hd-zip protein 8 homeobox-leucine zipper protein athb-15 hypothetical protein
VIT_210s0003g04670	*HOX32*	380-541	161	1.56E-13	homeobox-leucine zipper protein hox32-like

The Grapevine Genome CRIBI Biotech website (http://genomes.cribi.unipd.it/grape/search_form.php) was used to identify the target gene name, grape ID, coordinates and their functions.
