# Supplementary material for: Characterization of VvmiR166s-Target Modules and Their Interaction Pathways in Modulation of Gibberellic-Acid-Induced Grape Seedless Berries
Source: Int J Mol Sci. 2023 Nov 14;24(22):16279. doi: 10.3390/ijms242216279 (PMC10670991; doi:10.3390/ijms242216279)
Supplement: Supplementary file 1 [file ijms-24-16279-s001.zip › Table S3.pdf]

Table S3 Sequences of primer

Genes primers	Forward primer sequences (5′–3′)	Reverse primer sequences (5′–3′)
166a qRT-PCR	AGGAAAGCTTGGTGTCAGGA	GGAAAGCGGGTGTTTGGAAT
166b qRT-PCR	TTTGAGGGGAATGTTGGCTG	CCTAGATGGCCGTGACTGAA
166c qRT-PCR	AGATCAAGCCCGAGAAAAGC	TCTGGCTCGAGGACACTTAC
166d qRT-PCR	CGATTGGGGAGCAGTGATTG	GAGGCCACCAACCATCTTTG
166e qRT-PCR	AATGTTGTCTGGCTCGAGGA	GATCAGCAGCAATGGAAGCA
166f qRT-PCR	GTTGTGCTGTGTGTGGTGTA	AAGAAGAGGAGGAGGAGGGT
166g qRT-PCR	TCCGGCATGCAAATTGAAGT	GGGGAATGTTGTCTGGTTCG
166h qRT-PCR	GGAAGGTTGAGAAGTTGAGCC	ATCTCGGACCAGGCTTCATT
HOX32 qRT-PCR	TTACAGAAGCAGGTCTCGCA	GGTTTTGCTGTTGTTGGTGC
HB-15 qRT-PCR	CTCTGCTCTGAGTTCCCACA	AGCATTGTCCTCTTCGGTCA
REVOLUTAqRT-PCR	AGGGTTTTGCTTACTTGCCG	AAGCAAGGCAATGGACAGTG
