# Supplementary material for: Characterization of VvmiR166s-Target Modules and Their Interaction Pathways in Modulation of Gibberellic-Acid-Induced Grape Seedless Berries
Source: Int J Mol Sci. 2023 Nov 14;24(22):16279. doi: 10.3390/ijms242216279 (PMC10670991; doi:10.3390/ijms242216279)
Supplement: Supplementary file 1 [file ijms-24-16279-s001.zip › Table S4.pdf]

Table S4 Subcellular vector construction primer

Gene Name	Forward primer	Reverse primer
VvATHB15	**GAAGATCT**ATGATGGCGGTTAGTTCGAG	**GGACTAGT**AACGAAAGACCAGTTGATGAAC
VvREV	**GAAGATCT**ATGGCGATGGCGATTGCGCAGCAG	**GGACTAGT**CACAAAAGACCAGTTGATGAAAGA
VvHOX32	**GAAGATCT**ATGGCGCTTTCTATGCAC	**GGACTAGT**CACAAAGGACCAGTTTAT

Table footnote: Bold font is the enzyme cleavage site. Red bold font is the enzyme cleavage site.
