# Supplementary material for: Characterization of VvmiR166s-Target Modules and Their Interaction Pathways in Modulation of Gibberellic-Acid-Induced Grape Seedless Berries
Source: Int J Mol Sci. 2023 Nov 14;24(22):16279. doi: 10.3390/ijms242216279 (PMC10670991; doi:10.3390/ijms242216279)
Supplement: Supplementary file 1 [file ijms-24-16279-s001.zip › Table S5.pdf]

Table S5 VvMIR166s and VvATHB15 PCR amplification primers

Gene Name	Forward primer	Reverse primer
VvMIR166a	**CATGCCATGG**AGAGGGAGATAGAAGACTTGAGTG	**GGACTAGT**TTATAATCGATCATCTTAGGTATATGG
VvMIR166b	**CATGCCATGG** TCCCCATCTCTCCAGCTC	**GAAGATCT** AGAGAGAAAGAGGGTTTGCCATT
VvMIR166c	**GAAGATCT**ATAACCCCCAAATCATTCTCTTC	**GGACTAGT**TCACTTCACCATTACACCAGTCT
VvMIR166d	**CATGCCATGG**TTGTCCATTTTCCACTCTTTTTC	**GAAGATCT**GTTCAAAACACAGTCAAAACTTCTC
VvMIR166e	**CATGCCATGG** TCACTGACCCAATTCCAAAC	**GGACTAGT** ATCTGCTCATGCCCAACC
VvMIR166f	**CATGCCATGG** ACCGCCACAACTCTATTCAA	**GAAGATCT** CCCATCCCATATCTCCCATA
VvMIR166g	**CATGCCATGG**ACAAAGGAAATTCCCATAAACTACAT	**GAAGATCT** GCCTCTTGCTGTTCCTGTG
VvMIR166h	**CATGCCATGG** GCCAAACACAGCCTCAGC	**GAAGATCT** GCTGCAGATTGCCTCCTC
VvATHB15	**GAAGATCT**ATGATGGCGGTTAGTTCGAG	**GGACTAGT**TCAAACGAAAGACCAGTTGATGAAC

Note: Bold font is the enzyme cleavage site. Red bold font is the enzyme cleavage site.
